# Development and validation of assessment of psycho-education of carers questionnaire: An early experience

**DOI:** 10.12669/pjms.345.15710

**Published:** 2018

**Authors:** Imran Ijaz Haider, Farah Tiwana, Noor Zohra, Khaleeq ur Rehaman

**Affiliations:** 1 Prof. Dr. Imran Ijaz Haider, FRCPsych (London) Department of Psychiatry and Behavioral Sciences, Fatima Memorial Hospital College of Medicine and Dentistry, Lahore, Pakistan; 2 Ms. Farah Tiwana, MSc Mental Health Studies. Department of Psychiatry and Behavioral Sciences, Fatima Memorial Hospital College of Medicine and Dentistry, Lahore, Pakistan; 3 Ms. Noor Zohra, PhD Scholar. Department of Human Development and Family Studies, Govt. College of Home Economics, Lahore, Pakistan; 4 Prof. Dr. Khaleeq ur Rehaman, FECSM (European Joint Committee). Department of Urology and Andrology, Fatima Memorial Hospital College of Medicine and Dentistry, Lahore, Pakistan

**Keywords:** Mental illness, Psycho-education, Caregivers, Internal consistency

## Abstract

**Background and Objective:**

Psycho-education is an intervention integrating psychotherapeutic and educational strategies. Whilst carer psycho-education is known to aid in psychiatric disorders, at present there is no known tool to assess the degree to which this is routinely provided by mental health professionals. Our objective was to develop and validate a tool, in English, which assesses psycho-education of carers of psychiatric patients in Pakistan.

**Methods:**

A questionnaire was generated in English. It was pretested on twenty male and female carers and was refined to attain a more reliable version. Sixty bilingual male and female primary carers, who were fluent in English, and had been in a care-giving role for more than three months were requested to complete the developed Questionnaire for the Assessment of Psycho-Education of Carers (APEC) at Fatima Memorial Hospital Psychiatry Out-patient department within a period of four months from December, 2017 to April, 2018. Carers were identified via patients presenting to a psychiatric OPD. Responses were analyzed for reliability and test retest consistency using Cronbach’s alpha analysis, Intraclass correlation coefficients, factor analysis and Paired *t*-test.

**Results:**

APEC was found to be easily understandable and capable of adequately assessing aspects of psycho-education. A high degree of internal consistency was demonstrated on cronbach’s alpha analysis. Cronbach’s α coefficient for various domains was sufficiently high ranging from0.76 to 0.960. Similarly, domains of (APEC) were highly correlated. Test-retest reliability was assessed by computing the correlation between Visits 1 and 2 scores.

**Conclusion:**

The developed questionnaire can adequately assess psycho-education of primary carers in mental health settings.

## INTRODUCTION

Psycho-education is a standard therapeutic intervention for common mental health problems.^1^ It refers to systemic and instructional provision of information to patients and carers, about illness and treatment, usually by healthcare professionals.^2^

Psycho-education was originally developed within the context of family therapy.^3^,^4^ It was theorized that mental illness and relapse were due to high ‘expressed emotions’ between family members and patients, such as; hostility, critical comments and emotional over-involvement.^5^ Psycho-education was developed as a strategy to help carers and patients understand psychiatric illnesses and support treatments. Ensuing research has consistently demonstrated a predictive relationship between high levels of expressed emotion and poorer mental health outcomes.^6^,^7^

The primary aim of psycho-education is the “empowerment of the affected”^8^ through the following steps: *Information Transfer*: symptomatology, aetiology and treatments. *Emotional Discharge*: expression of emotions in the context of illness. *Support of Medication or Psychotherapy*: cooperation between health professional and carer/patient. *Assistance to Self Help*: Encouraging patient/carer to develop autonomy.

Psycho-education is helpful for carers and sufferers of conditions such as Post Traumatic Stress Disorder^9^, Schizophrenia^10^, Bipolar Affective Disorder^11^ and Major Depression.^12^ Additionally, local psycho-educational programmes for Schizophrenia show reduced relapse rates, and better coping skills.^13^

Psycho-education can positively impact treatment adherence^14^, and the experiences of mental health care by patients and their families. Carer psycho-education helps reduce the distress of caring for a person with mental illness^15^ and improves quality of life and care-giving experience.^16^

Unlike patient psycho-education, which is relatively easy to evaluate because it is shared routinely, carer psycho-education may be neglected with patients as the primary focus of healthcare professionals. At present, there are no empirically validated instruments to assess carer psycho-education. Without a validated self-reporting instrument that objectively measures psycho-education, outcome research in this area will continue to be limited. Hence, the present study was undertaken to develop and validate a brief self-reporting measure of carer psycho-education covering key domains.

## METHODS

We have developed a questionnaire for use by mental health professionals and researchers to enable assessment of carer psycho-education. The study was approved by Institutional Review Board of Fatima Memorial Hospital College of Medicine and Dentistry. In phase I, Assessment of Psycho-Education of Carers questionnaire (APEC) was developed (Questionnaire Supplementary file available online). A panel of experts including Psychiatrists, Urologist, Psychologists and Researchers generated the items related to psycho-educational domains. These were originally 19, modified to 12 after discussion.

Basic psychometric criteria were applied, i.e., clarity and understandability, comprehensive understanding of response choices and relative simplicity of administration and scoring. The questionnaire was filled by a sample of twenty-two primary carers. Majority participants understood questions and response options; nonetheless some questions were rephrased for further clarification. Experts reviewed feedback and provided additional input.

In phase II, the 12-item questionnaire resulting from Phase I testing was administered to 60 participants, recruited from amongst carers of psychiatric OPD patients at Fatima Memorial Hospital, Lahore. The study took place for approximately four months from 19 December 2017 to 30 April 2018 at the Psychiatry Department of Fatima Memorial Hospital.

Inclusion criteria comprised individuals who were bilingually educated, and were primary carers of patients suffering from mental illnesses. Carers were aged 18 and above and were all educated and fluent in English. Prior to questionnaire administration, oral and written informed consent was taken from each participant. Informal demographic data were collected regarding carers’ age, gender, education, relationship to patient and average daily time spent with patient. On average, carers spent up to six hours daily with patients.

APEC was examined for construct validity and reliability. It was administered twice (Visit 1 and Visit 2), two to four weeks apart, to assess test-retest reliability. At Visit 1, participants also completed a demographic data form as an assessment of divergent validity. The first series of evaluations were performed on an item-by-item basis in order to obtain items with adequate psychometric properties and clinical relevance for the final questionnaire. Factor analysis was performed to assess underlying domain structure of the questionnaire, and to evaluate factorial validity. Internal consistency of items within each factor was evaluated using Cronbach’s alpha. Test-retest reliability was determined by means of a t-test. In the final series of analyses, psychometric properties of the questionnaire resulting from the above item-reduction process were fully evaluated.

The scoring appendix of APEC questionnaire is as follows: Domain I represents “Nature of the illness” (score range 3-12), domain II represents “Satisfaction and benefit of the information provided by the mental health professional” (score range 3-12), Domain III represents “Information about medications” and its sub domain”3a” represents “Information about use of medications” (score range 3-12) and sub domain”3b” represents “Information about side effects of medications” (score range 3-12) respectively. The lowest to highest score ranges from 15-60.

Participants completed the self-reporting questionnaire in English and were then informally interviewed by the researchers, and responses were matched with answers to the questionnaire. Participants responded to four point likert-scale options ranging from *not aware (1)* to *full aware (4)*.

The current questionnaire was developed in English to allow participation from a broader range of individuals. Once we have completed this task we aim to translate this questionnaire to local languages i.e. Urdu, Punjabi, Sindhi and Pashto.

## RESULTS

Cronbach’s α coefficients were determined for total and domain scores of the questionnaire which were significantly high, ranging from 0.760 to 0.960 for the entire sample of sixty participants indicating that the questionnaire has good internal consistency and reliability. Individual domain scores were calculated by adding the scores of the individual items that comprise the domain. The full-scale score was calculated by adding the three domain scores. The 12-item questionnaire APEC was assigned with four factors that corresponded to domains “nature of illness, satisfaction and benefit of information, information about use of medication and its side effects” with eigenvalues of 4.696, 2.157, 2.132 and 1.199 respectively. These four factors accounted for 84.874% of explained variance and the lowest eigenvalue was 1.199. Factor 4 has less loading than 0.5 (Table-II).

**Table-I:** Mean values and cronbach’s α coefficient of the questionnaire for assessment of psycho-education of carers(APEC) (n = 60).

Domain name	Mean ± SD	Cronbach’s alpha	Number of items
**Domain1:** About nature of the illness	2.36±0.879	0.760	3
a. Name of illness	2.18±1.24		
b. Common signs and symptoms	2.47±0.91		
c. Progress of the illness	2.43±1.03		
**Domain 2:** Satisfaction & benefit of information provided	2.41±1.056	0.960	3
e. Was information provided understandable?	2.42±1.12		
f. Satisfaction about information provided	2.40±1.06		
g. Was information provided was helpful	2.43±1.11		
**Domain 3a:** Information regarding use of medications	2.66±0.872	0.919	3
k. Information about use of medicines prescribed	2.62±0.922		
l. Information how many times this medication is to be taken	2.80±1.005		
m. Satisfaction about the information regarding use of medications	2.58±0.889		
**Domain3b:** Information regarding side-effects of medications	1.488±0.709	0.902	3
n. Information about the side effects of medications prescribed	1.47±0.769		
o. Information what to do in case of side effects of medications	1.43±0.767		
p. Satisfaction about the information provided regarding the side effects of the medications	1.57±0.789		
Full scale scores	26.8±7.28	0.854	12

The individual domain scores were calculated by adding the scores of the individual items that comprise the domain and multiplying the sum by domain factor. †The full-scale score is calculated by adding the three domain scores.SD = standard deviation.

**Table-II:** Factor analysis of the Questionnaire for Assessment of psycho education of carers APEC (n = 60)

Factors				
**Domain names**	**1**	**2**	**3**	**4**
a. Name of illness	0.291	0.160	0.301	**0.768**
b. Common signs and symptoms	0.113	0.295	0.069	**0.860**
c. Progress of the illness	0.289	-0.086	-0.058	**0.699**
d. Was information provided understandable	**0.910**	0.065	0.075	0.248
e. Satisfaction about information provided	**0.908**	0.172	0.173	0.195
f. Was information provided was helpful	**0.946**	0.095	0.074	0.225
g. Information about use of medicines prescribed	-0.066	**0.933**	-0.016	0.125
h. Information how many times this medication is to be taken	0.182	**0.899**	0.129	0.066
i. confident about the information regarding use of medications	0.196	**0.907**	0.105	0.097
j. Information about the side effects of medications prescribed	0.014	0.060	**0.864**	0.125
k. Management in case of side effects	0.093	0.014	**0.922**	0.156
l. confident about the information provided regarding the side effects of the medications	0.185	0.135	**0.920**	-0.083
EigenValues	4.696	2.157	2.132	1.199
Percentage of explained variance	39.135	17.977	17.768	9.994

Extraction method: principal component analysis. Rotation method: varimax with Kaiser normalization. *The highest factor loading in each principal component are shown in bold. Kaiser–Meyer–Olkin measure of sampling adequacy = 0. 752, which is <0.7. Furthermore, Bartlett test of sphericity (χ2 =577.409) was calculated.

Test-retest reliability was assessed by the stability coefficient between Visits 1 and 2 scores. As seen in Table-III, overall test-retest reliability was relatively high for all of the domains (r = 0.980to 0.999) and for the total scale (r = 0.996). In APEC, domain II showed the highest test-retest reliability (r =0.999).

**Table-III:** Test–retest mean scores and paired sample *t*-test (n = 22).

Mean Differences								

				95% confidence interval of the difference				

Domains	Test mean (SD) n = 22	Retest mean (SD) n = 22	Mean difference SD	Lower	Upper	*t*	df	*P* value
Nature	2.12(0.82)	2.18(0.77)	-0.060(0.22)	-0.158	0.037	-1.28	21	0.213
Satisfaction and benefit	2.27(0.98)	2.25(0.95)	0.015(0.071)	-0.016	0.046	1.00	21	0.329
Medication use	2.57(0.68)	2.53(0.74)	0.045(0.155)	-0.023	0.114	1.36	21	0.186
Side effects of medication	1.36(0.58)	1.33(0.57)	0.03(0.098)	-0.013	0.073	1.44	21	0.162
Full scale	2.08(0.54)	2.07(0.53)	0.007(0.067)	-0.022	0.037	0.52	21	0.605

df = degrees of freedom; SD = standard deviation.

## DISCUSSION

Our objective was to develop a brief, valid, and reliable self-report measure of Assessment of Psycho-Education of Carers which could be easily administered to primary caregivers across a wide range.

The advantage of APEC is that it assesses carer psycho-education. To our knowledge, at present there is no other validated tool for this purpose. An expert panel concluded that the inclusion of mentioned domains will provide evidence whether psycho-education is being effectively provided.

A limitation of APEC is its sole focus on carers and their psycho-education. Furthermore, carers unable to understand English and those who were illiterate were excluded, thereby limiting the reach of APEC. It provides a broad measure of psycho-education across three domains, and is equally applicable for researchers and clinicians.

Psycho-education improves quality of life, functionality and boosts positive outcomes for patients and families.^17^ APEC will encourage regular psycho-education catering to specific carer needs and will allow clinicians to assess effectiveness and understandability of psycho-education. Such a questionnaire will also promote support for carers and recognition of their needs and challenges.

“Domain II” addressed satisfaction with and benefits from information provided. According to research, carer-clinician agreement regarding prognosis communication is no greater than patient–clinician agreement.^18^ APEC will allow for a better flow of communication between carers and clinicians.

“Domain III” considered medication use. Carers’ negative emotional states, physical impairment and low literacy are hindrances in medication management.^19^ Withthis awareness, clinicians can use APEC to further modify psycho-education provided according to the carers emotional needs and their educational status.

It’s about sub-domains 3b considered information regarding side effects and their management. The frequently overlooked need to support to carers can significantly affect patient recovery.^20^ Knowledge of medications, side-effects and their management will improve adherence and a create positive experience for the carer.^21^

This will help psychiatrists, psychologists and researchers as well as carers and patients. In Pakistani society, the bulk of caregiving burden falls on the eldest member of the family or closest blood relative. Therefore, psycho-education is an imperative need and right of patients and carers. Use of such a questionnaire is not limited to research but is equally applicable to clinical practice to bridge communication between carers and health care professionals and allow for an authentically collaborative approach to treatment and recovery.

## CONCLUSIONS

The APEC, a 12-item questionnaire, has been developed as a brief, multidimensional self-report instrument for assessing the key dimensions of psycho-education. It is psychometrically sound, and easy to administer. The questionnaire described was designed and validated for assessment of psycho-education of psychiatric carers. It can be used for research on the literate population of Pakistan.
